# Intraoperative electrocortical stimulation of Brodman area 4: a 10-year analysis of 255 cases

**DOI:** 10.1186/1746-160X-2-20

**Published:** 2006-07-03

**Authors:** Olaf Suess, Silke Suess, Mario Brock, Theodoros Kombos

**Affiliations:** 1 Department of Neurosurgery, Charité – Universitaetsmedizin Berlin, Campus Benjamin Franklin, Berlin, Germany

## Abstract

**Background:**

Brain tumor surgery is limited by the risk of postoperative neurological deficits. Intraoperative neurophysiological examination techniques, which are based on the electrical excitability of the human brain cortex, are thus still indispensable for surgery in eloquent areas such as the primary motor cortex (Brodman Area 4).

**Methods:**

This study analyzed the data obtained from a total of 255 cerebral interventions for lesions with direct contact to (121) or immediately adjacent to (134) Brodman Area 4 in order to optimize stimulation parameters and to search for direct correlation between intraoperative potential changes and specific surgical maneuvers when using monopolar cortex stimulation (MCS) for electrocortical mapping and continuous intraoperative neurophysiological monitoring.

**Results:**

Compound muscle action potentials (CMAPs) were recorded from the thenar muscles and forearm flexors in accordance with the large representational area of the hand and forearm in Brodman Area 4. By optimizing the stimulation parameters in two steps (step 1: stimulation frequency and step 2: train sequence) MCS was successful in 91% (232/255) of the cases. Statistical analysis of the parameters latency, potential width and amplitude showed spontaneous latency prolongations and abrupt amplitude reductions as a reliable warning signal for direct involvement of the motor cortex or motor pathways.

**Conclusion:**

MCS must be considered a stimulation technique that enables reliable qualitative analysis of the recorded potentials, which may thus be regarded as directly predictive. Nevertheless, like other intraoperative neurophysiological examination techniques, MCS has technical, anatomical and neurophysiological limitations. A variety of surgical and non-surgical influences can be reason for false positive or false negative measurements.

## Background

Tumor invasion in functional cortex areas, tumor-related mass displacements and functional cortical reorganization can greatly impede intraoperative orientation in eloquent areas of the brain, such as the primary motor cortex (Brodman Area 4). Intraoperative neurophysiological examination methods are nowadays thus indispensable for surgery in or near the motor cortex [1-7].

Many techniques have been developed for direct electrical stimulation of motor pathways [8-13]. Towards the end of the 19^th ^century, Sir Victor Horsley [10,11] had already published several studies describing movements triggered in the extremities of monkeys by electrically stimulating the cortex. In the course of the following decades, modifications of this technique and their application in awake operated humans were described by various authors, including Gruenbaum and Sherrington in 1903 [9] as well as Cushing in 1909 [8]. However, it has taken several decades for direct cortical stimulation to be applied clinically. A study by Penfield and Boldrey in 1937 [12] finally laid the foundation for establishing specific intraoperative neurophysiological mapping and monitoring techniques.

The methodology for eliciting MEPs intraoperatively has its origin in the early works of Patton and Amassian [14]. They were able to demonstrate that direct electrical stimulation of the motor cortex generates a series of descending volleys in the pyramidal tract, which could be easily recorded over the exposed pyramids of the medulla.

It was only in the year 1990 that Berger et al. [7] described a modification of that bipolar technique already used by Penfield. This modification enabled direct electrical cortex stimulation even during surgery under general anesthesia. Although this method does not allow qualitative analysis of the mass movements it evokes, this bipolar stimulation technique has since been regarded as the standard method of intraoperative cortex stimulation.

The choice of a monopolar stimulus for direct cortical stimulation is partially based on investigations by Hern in the early sixties [15], who described the direct electrical excitability of pyramidal cells of the motor cortex of baboons and was first to propagate an anodal stimulation technique for this purpose. Rank [16] later performed a series of electrophysiological investigations in mammals showing that anodal high-frequency stimulation leads to direct excitation of the axons of pyramidal cells. In 1993, Taniguchi et al. [17] described a modification of this monopolar stimulation technique for the intraoperative application in human brain surgery. Using a high-frequency anodal square-wave pulse, compound muscle action potentials (CMAPs, a group of almost simultaneous action potentials from several muscle fibers in the same area) were evoked by stimulation of the supplying cortical motor area and are recorded as one multipeaked summated action potential in muscles of the contralateral extremities during surgery under general anesthesia. This is done via direct excitation of Betz's pyramidal cells in the fifth layer of the six-layered isocortex exited by the fast-conducting thickly myelinized pyramidal fibers. These originate from the motor cortex areas and pass through the corona radiata and the posterior limb of the internal capsule. They then cross the middle part of the cerebral peduncle as well as the pons and extend to the base of the medulla oblongata. The pyramidal decussation at their lower end is where approximately 85% of the fibers cross to the opposite side [18]. The fibers crossing at medullary level pass downwards through the lateral white column of the spinal cord in the so-called lateral corticospinal tract and end segmentally at the α horn cells or γ motor cells. From there, α and γ fibers extend to the motor endplates of the respective muscles, where a compound muscle action potential can then be recorded with the aid of subdermal needle electrodes. This multi-pulse technique essentially differs from Penfield's technique in that it calls for only 5–7 stimuli with up to 500 Hz of stimulation rate, while Penfield's technique calls for continuous stimulation during a few seconds with a frequency of 50–60 Hz.

Several studies have since described the basic applicability of this monopolar procedure for intraoperative neurophysiological monitoring of the motor cortex [4,6,19,20]. The study presented here was performed to examine whether repetitive monopolar stimulation is possible throughout the entire course of a surgical procedure not only as a mapping but also as a monitoring technique, whether an optimization of the stimulation parameters can increase the success rate of positive stimulations and whether changes in the recorded CMAPs can be correlated directly with surgical maneuvers or other non-surgical influences as well as with the specific postoperative neurological symptoms.

## Methods

### Patients

**Figure 1:**
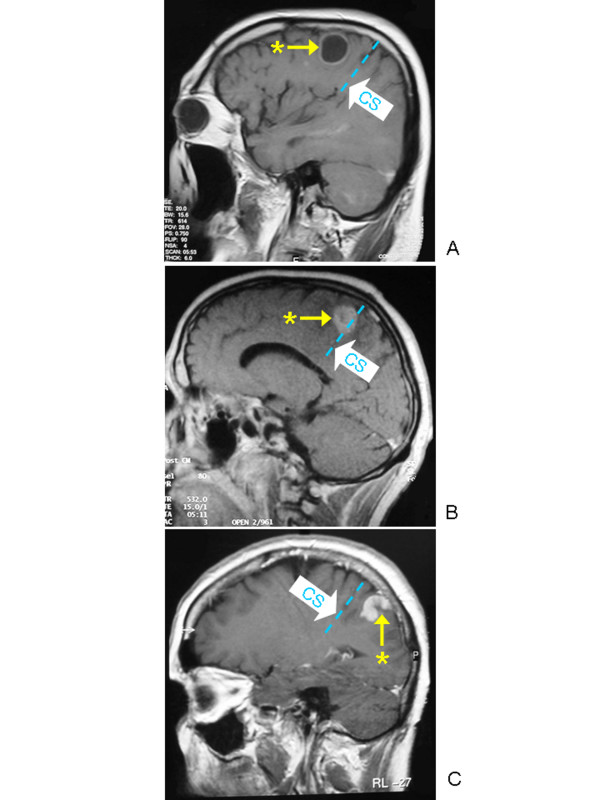
Gadolinium enhanced T1 weighted sagittal MR images showing examples of lesions that are located frontal to Brodman Area 4 (**A**), dorsal to Brodman Area 4 (**C**) or had direct contact with Brodman Area 4 (**B**). CS = central sulcus; * = tumor.

Over a period of 10 years (January 1996 to January 2006) 255 patients undergoing surgery in or immediately adjacent to Brodman area 4 were intraoperatively submitted to MCS for both mapping and monitoring of motor function. There were 137 women and 118 men with a mean age of 57.3 years (16–87 y.). The topographic relationship between the lesion and Brodman area 4 was evaluated preoperatively by means of CT or MRI. One hundred seventeen lesions were in the non-dominant hemisphere, whereas 138 were in the dominant. They were located frontal to Brodman Area 4 in 89 cases (Figure 1A), dorsal to Brodman Area 4 in 55 cases (Figure 1C) and had direct contact with Brodman Area 4 in another 111 cases (Figure 1B). Histological diagnosis included metastases (107), gliomas WHO IV (69), gliomas WHO II–III (44), meningiomas (12), arteriovenous malformations (7), oligodendrogliomas (5), cavernomas (5), gliosarcomas (2), PNET (2), chondroma (1) and epidermoid cyst (1) (Table 1). All patients underwent a pre- and postoperative clinical evaluation according to a standardized protocol. Muscle strength was graded according to the British Medical Research Council Scale.

**Table 1:** Age and gender distribution, localization of the lesions and histological diagnosis of 255 cases.

Age and sex distribution:	
Men: 118/women: 137	n = 255
Age: 16–87 years	Mean age: 57.3
	
Localization:	
Dominant hemisphere: 138	Nondominant hemisphere: 117
	
Frontal contact with area 4	89
Direct involvement of area 4	111
Dorsal contact with area 4	55
	
Histological examination results:	
Metastases	107
Gliomas WHO IV	69
Gliomas WHO II–III	44
Meningiomas	12
Arteriovenous malformations	7
Oligodendrogliomas	5
Cavernomas	5
Gliosarcomas	2
PNET	2
Chondroma	1
Epidermoid cyst	1

### Anesthesia

In all 255 cases, intravenous anesthesia (TIVA) was performed without administering volatile anesthetics. Induction of anesthesia was achieved by a bolus of propofol (1–2 mg/kg) and fentanyl (5–10 μg/kg). Anesthesia was maintained by continuous propofol administration (75–125 μg/kg/h). Intraoperative analgesia was carried out with fentanyl (1–2 μg/kg/h). Neuromuscular blocking agents were used only for intubation (rocuronium 0.3–0.4 mg/kg or mivacurium 0.2 mg/kg) but not during surgery. With this setup, neuromuscular blocking was effective for only 15–25 min during intubation and TOF-monitored patient positioning. No further muscle relaxants or drugs with a muscle-relaxing side effect were used in the course of the operation.

### Intraoperative setup

**Figure 2:**
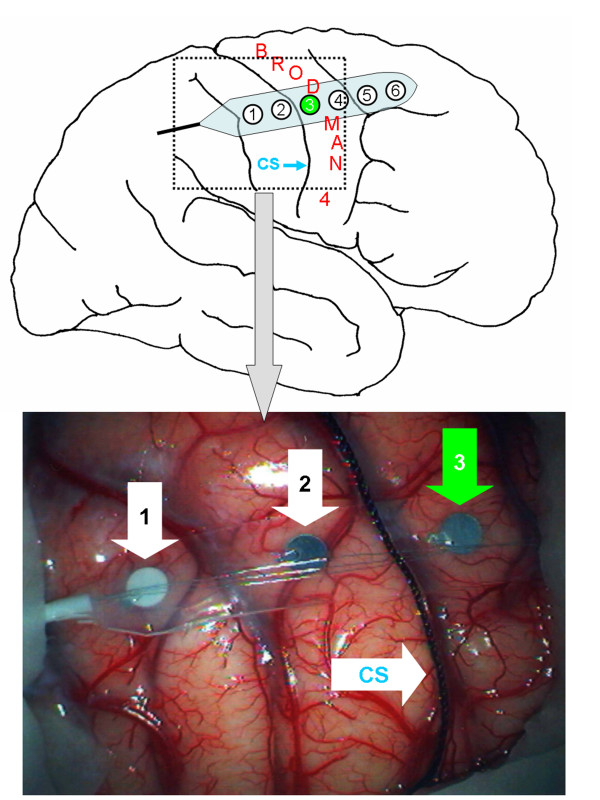
Placement of a 6-contact strip electrode on the cortex. Electrode No. 3 is placed directly over Brodman Area 4. CS = central sulcus.

After opening the dura, a 6-contact strip electrode (AD-Tech^® ^strip electrode, AD Technic, WI, USA) was placed on the exposed cortex at an approximately 65° angle to the sulcus relief (Figure 2). In each case, one of the contact electrodes was used as the anode, while an adhesive electrode (Neuroline^® ^Disposable Electrode Type 710 15-K, Ambu Medicotest A/S, Denmark) attached to the ipsilateral frontal region (Fp1 or Fp2 according to the 10–20 International System) served as the cathode. All measurements were performed with a Nicolet Viking IV™ or Endeavour™ (Viasys Healthcare/Nicolet Biomedical, Madison, WI, USA).

Intraoperative identification of the central sulcus and Brodman area 4 was made using a combination of somatosensory evoked potential phase reversal and direct monopolar anodal high-frequency electrical stimulation of the cortex [4,20,21]. The basic setting selected for direct cortex stimulation was a monopolar square-wave pulse with a duration of 0.3 ms, a stimulation frequency of 400 Hz and a sequence (train) of 5 pulses. The stimulation intensity was increased in 1 mA steps, starting from the zero position, until a muscle action potential could be recorded or an upper limit of 25 mA was reached. If no CMAP could be triggered at this setting, the stimulation frequency was increased to 500 Hz. In case of renewed failure, the pulse sequence was increased from 5 to 7 pulses.

**Figure 3:**
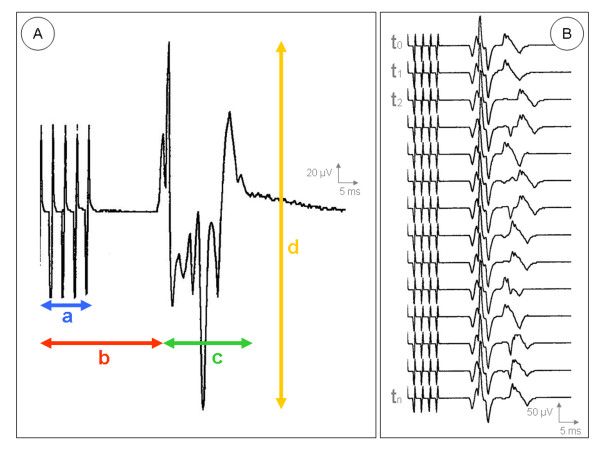
(**A**) Individual compound muscle action potential (CMAP) with a: stimulation artifact; b: latency (in ms); c: potential width (in ms) and d: amplitude (in μV). (**B**) The potential curve for course monitoring, starting with an individual basal value (t_0_), is registered on a separate time axis.

Motor responses were recorded by subdermal needle electrodes attached in a bipolar setup. Using a standardized protocol, disposable monopolar needle electrodes (20 mm/28 gauge or 25 mm/27 gauge) were placed 5 – 10 mm apart over characteristic muscle groups such as the thenar muscles (abductor muscle of the thumb), forearm flexors (ventral side of forearm, halfway between the wrist and the elbow over the radial flexor muscle of the wrist, long palmar muscle, superficial flexor muscle of the fingers and ulnar flexor muscle of the wrist), the quadriceps femoris muscle (halfway between the anterior superior iliac spine and the patella) and the gastrocnemius muscle on the contralateral side of the body. For recording, filters were set at 100 Hz to 10 kHz and sensitivity at 100 μV to 1 mV. The time base was 20 to 500 msec. The motor responses (compound muscle action potentials – CMAPs) were continuously displayed on a monitor screen, analyzed online according to their latency, potential width and amplitude and stored on a hard disk for further offline analysis. The latency was considered to be the time span (in ms) from the beginning of the stimulation sequence to the first measurable potential deflection. The potential width was defined as the time span (in ms) between the first and last measurable potential deflection. The amplitude (in μV) was measured by selecting the height between the two amplitude peaks (peak-to-peak) of the greatest measurable potential deflection (Figure 3A).

**Figure 4:**
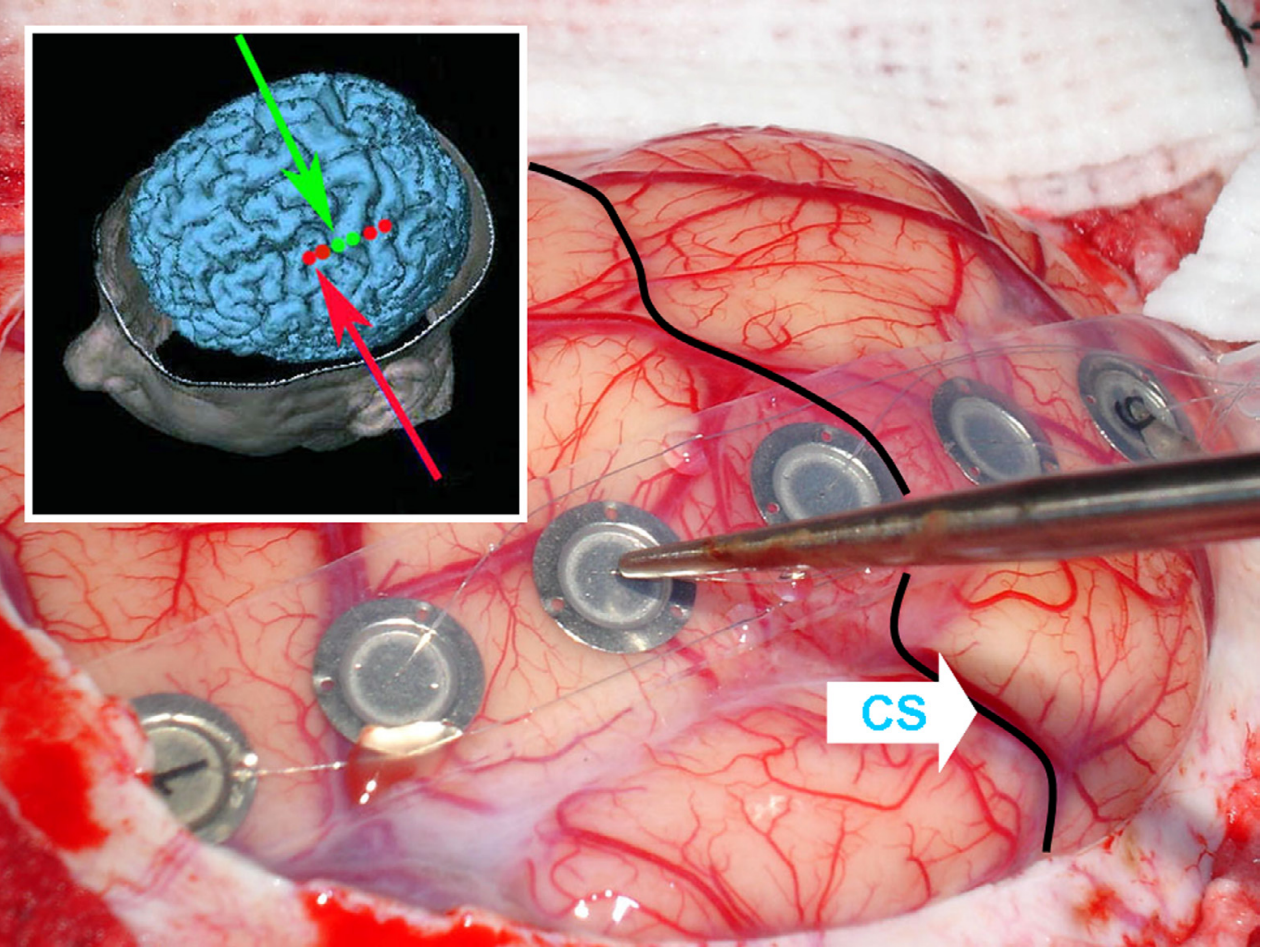
Screenshot of the navigation system with stimulation sites of the cortical 6-contact strip electrode visualized in the 3D brain surface model. stimulation sites in green = Motor cortex/Brodman Area 4; stimulation sites in red = no motor function; CS = central sulcus.

The central sulcus and the cortical points at which stimulation triggered a CMAP were marked on the cortex and photo documented. Since June 2002 the coordinates of the stimulation sites were additionally visualized and stored in 48 cases with the aid of a neuronavigation system (ACCISS II™, Schaerer Mayfield Technologies GmbH, Berlin, Germany) (Figure 4). This helped to better identify the stimulation sites and their anatomical localization compared to the precentral gyrus (Brodman Area 4) and the lesion to be removed.

For MCS monitoring an individual basal value (t_0_) was obtained at the cortical site that was used for repetitive stimulation during surgery. The potential curve for course monitoring was registered on a separate time axis on the screen of the monitoring device (Figure 3B). Depending on the operation phase, monitoring was performed at 30-seconds to 5-minute intervals and ended with a final measurement after tumor removal and closure of the dura. Potential changes were calculated as difference in percentage (+/- %) related to the t_0_-CMAP. Any intraoperative potential changes were immediately reported to the surgeon and correlated with the operative maneuvers performed shortly before.

## Results

### Stimulation parameters for electrocortical mapping

**Figure 5:**
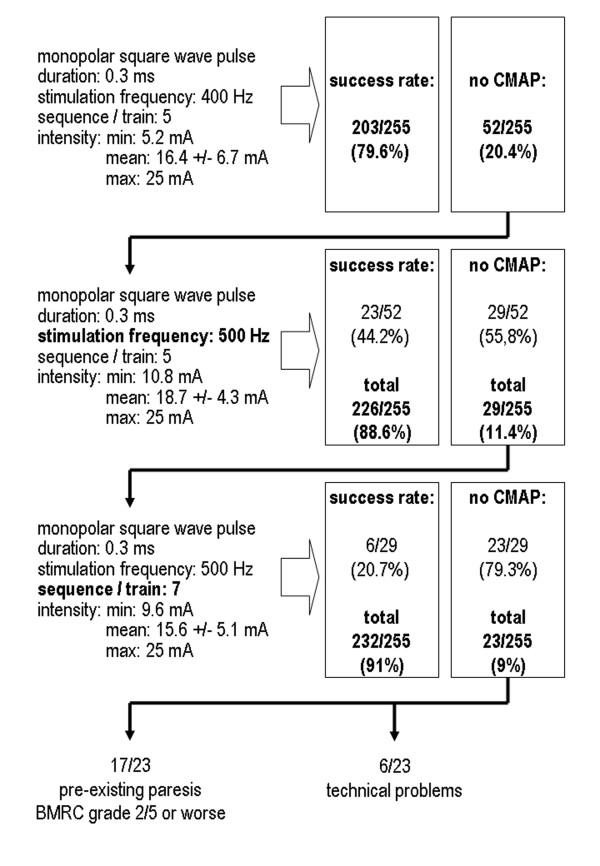
Diagram showing how the overall success rate could be improved by adapting e.g. the stimulation parameters frequency and train.

The mean stimulation intensity needed to trigger a CMAP under the basic setting (monopolar square-wave pulse with a duration of 0.3 ms, a stimulation frequency of 400 Hz and a sequence/train of 5 pulses) was 16.4 ± 6.7 mA (Figure 5). This enabled mapping of Brodman Area 4 in 203 of the 255 cases (79.6%). A muscle action potential could be triggered in another 23 cases (additional 9.0%) by increasing the stimulation frequency from 400 to 500 Hz. Increasing the impulse sequence from 5 to 7 pulses ultimately triggered a CMAP recordable via the contralateral extremity muscles in another 6 cases (additional 2.4%). Brodman Area 4 could thus be localized with the aid of MCS in a total of 232 of the 255 cases (91%). The 23 cases (9.0%) where no CMAP could be triggered by MCS involved 17 patients with pre-existent high-grade pareses (BMRC grade 2/5 or worse) and 6 cases with technical problems (3× defect of an electrode, 1× electrode displacement, 1× software problem, 1× defect of stimulator).

### Recording sites

An analysis of the different recording sites showed, that a CMAP could be recorded over the thenar muscles (TM) in 85.4% of the cases, over the forearm flexors (FF) in 68.4%, parallel over the TM as well as the FF in 54.3%, over the gastrocnemius muscle (GM) in 19.4%, the quadriceps muscles (QM) in 17.2%, and parallel over both the GM and QM in another 11.6% of the cases.

### Electrocortical monitoring

As already known from previous studies [4-6,20] with smaller groups of patients, CMAP recordings after MCS for continuous intraoperative monitoring are characterized by individual deviations of up to 5% for the latencies, 30% for the potential widths and 50% for the amplitudes without any pathological background. These individual deviations were characterized by inconstancy, i.e. "oscillation" around the initial value t_0_, and by the statistical correlation analysis showing independence from the related current intensity (n = 232 cases, 11856 CMAPs; 5.2 – 25 mA; r_latency _= -0.19; r_potential width _= -0.15; r_amplitude _= 0.09). However, there were potential changes that exceeded the above mentioned statistical scattering range and lacked the typical oscillating character or showed constant progression under repetitive stimulation. This was the case in a total of 47 of the 232 series of measurements.

**Figure 6:**
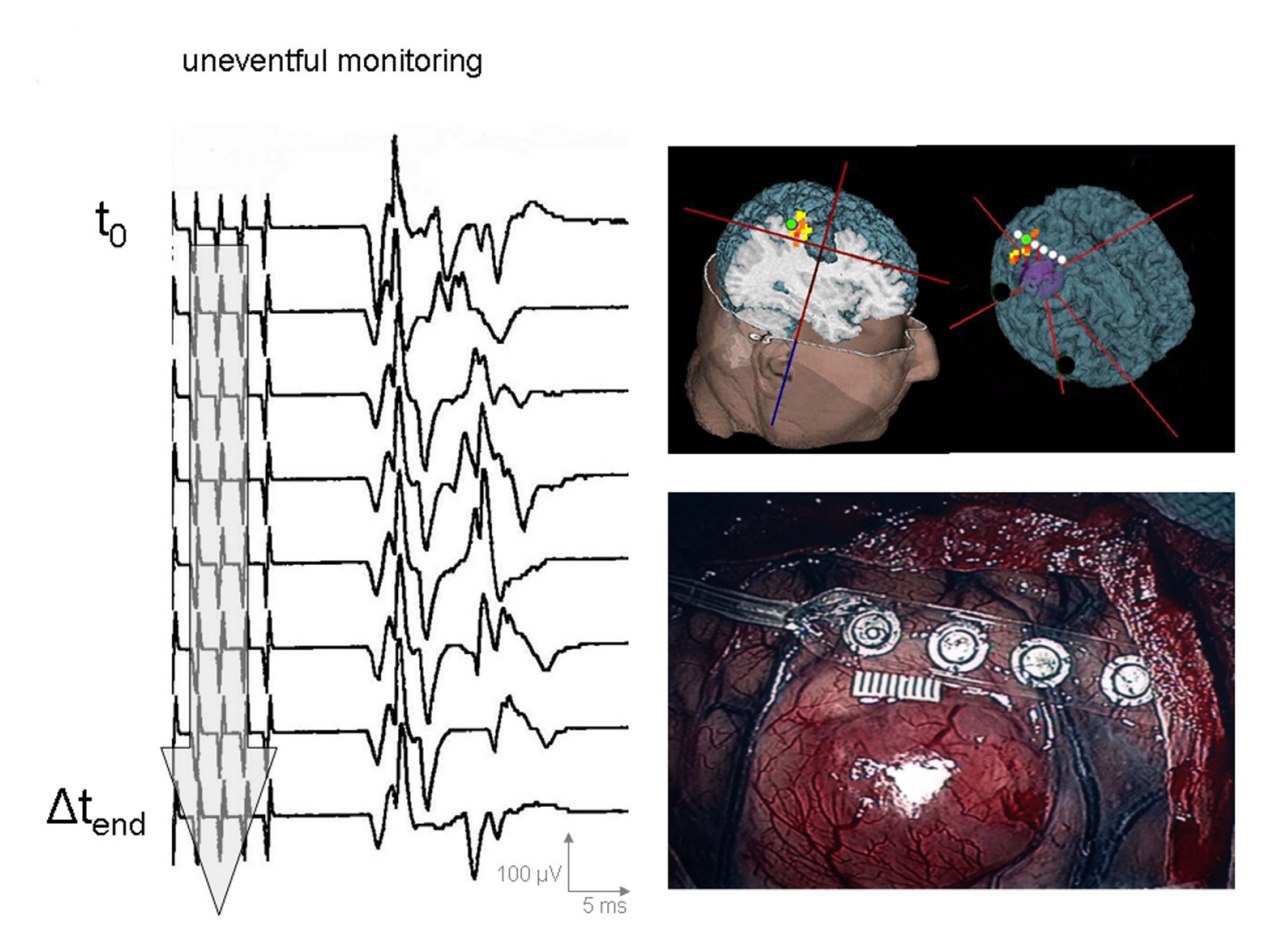
Illustrative case of *uneventful *MCS monitoring (Group A) in a patient with a right frontal metastasis. t_0 _= time of first stimulation; Δt_X _= onset of potential change; Δt_end _= end of tumor resection/last stimulation before dura closure; recordings from thenar muscle.

**Figure 7:**
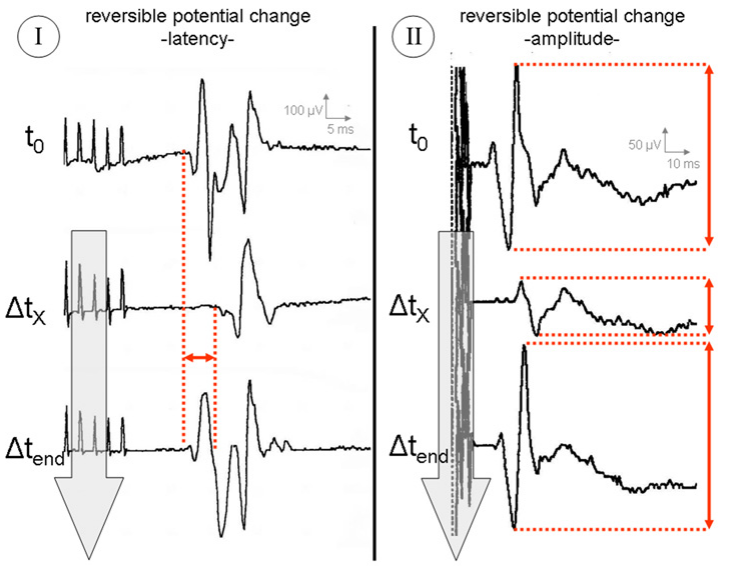
Illustrative cases of abnormal MCS monitoring with *reversible *potential changes (Group B). t_0 _= time of first stimulation; Δt_X _= onset of potential change; Δt_end _= end of tumor resection/last stimulation before dura closure; **I **= recordings from thenar muscle; **II **= recordings from forearm flexors.

**Figure 8:**
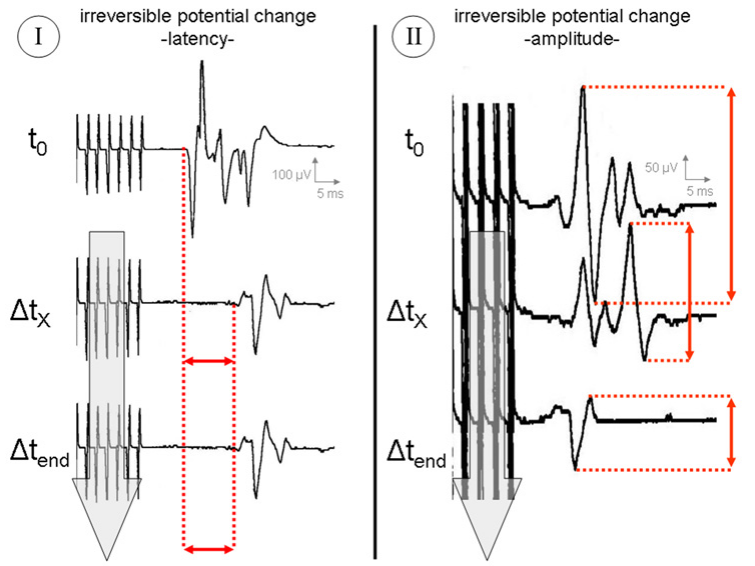
Illustrative cases of abnormal MCS monitoring with *irreversible *potential changes (Group C). t_0 _= time of first stimulation; Δt_X _= onset of potential change; Δt_end _= end of tumor resection/last stimulation before dura closure; **I **= recordings from thenar muscle; **II **= recordings from forearm flexors.

Three groups were ultimately differentiated (Table 2): *Group A:*Series of measurements with uneventful MCS monitoring characterized by individual CMAPs within a 5% range around the t_0_-latency, a 30% range around the t_0_-potential width and within a 50% range around the t_0_-amplitude (Figure 6); *Group B:*Series of measurements with potential changes exceeding the above mentioned ranges at least three times within 90 seconds, but with full reversibility until the end of the procedure (Figure 7) and *Group C:*Series of measurements with potential changes exceeding the above mentioned ranges at least three times within 90 seconds, but without any tendency of recovery until the end of the procedure (Figure 8).

**Table 2:** Correlation between potential changes detected intraoperatively during MCS monitoring and postoperative neurological symptoms. Total number and percentage of analyzed cases.

	Postoperative motor strength **improved **(according to the BMRC grading)	Postoperative motor strength **Unchanged**	Postoperative motor strength **deteriorated < 72 hours**	Postoperative motor strength **deteriorated >72 hours**	
**Group A:** MCS monitoring **uneventful,**	42 (18.1%)	131 (56.5%)	10 (4.3%)	2 (0.9%)	**185 (79.8%)**
**Group B:** MCS monitoring **abnormal, reversible,**	7 (3.0%)	14 (6.0%)	5 (2.2%)	1 (0.4%)	**27 (11.6%)**
**Group C:** MCS monitoring **abnormal, irreversible,**	0 (0%)	3 (1.3%)	0 (0%)	17 (7.3%)	**20 (8.6%)**
	**49 (21.1%)**	**148 (63.8%)**	**15 (6.5%)**	**20 (8.6%)**	**Σ = 232 (100%)**

#### Group A

In 185 of the 232 MCS monitoring cases (79.8%), no significant potential changes could be observed apart from the individual potential fluctuations previously described. One hundred thirty-one of these 185 cases had completely uneventful intraoperative monitoring and showed no postoperative change in neurological symptoms compared to the preoperative examination. In 42 cases tumor excision even led to clinical improvement of a preoperative paresis without any neurophysiological correlative. However, another 12 cases showed postoperative deterioration of pre-existent pareses or recurrence of unilateral symptoms. In 10 of the 12 cases, these were limited to the first 72 postoperative hours and correlated with postoperative perifocal brain edema on the CT image controls. Only two patients developed permanent brachial paresis after surgery (BMRC grade 1/5). In these cases, follow-up imaging disclosed an infarction involving Brodman Area 4.

#### Group B

In a total of 27 of the 232 cases (11.6%), the observed potential changes significantly exceeded the individual potential fluctuations previously described. Latency prolongations of > 5% and amplitude reductions of > 50% could be documented in at least 3 measurements within 90 seconds. However, threshold values for changes in potential widths could not be determined in the presence of a very inhomogeneous scattering range. A direct correlation was found for the following surgical maneuvers:

(a) traction or pressure by applying a brain spatula to the primary motor cortex (10/27),

(b) cold irrigation (7/27),

(c) electrocoagulation near the primary motor cortex or motor pathways (6/27) and

(d) displacement/shifting of the electrode strip (4/27).

Information to the surgeon meant interrupting all surgical maneuvers, releasing the spatula, stopping the electrocoagulation and checking the placement of the electrode strip. All 10 cases where brain spatula pressure or traction correlated with potential changes had a full recovery of potentials to the initial value t_0 _(+/- individual scattering range) within a maximum of 5 minutes (15 to 290 sec; mean: 3.1 min). The mean recovery time was 4.8 min (35 to 450 sec) in the cases of cold irrigation as source of the potential changes and 5.6 min (60 to 720 sec) in the cases attributed to electrocoagulation. After remission of the potential change, the operation was continued, taking into account the acquired functional and anatomic information. In the 4 cases of electrode displacement the strip electrodes were readjusted according to the anatomical landmarks or with the help of the spatial information of the neuronavigation system. The postoperative neurological examination showed unchanged neurological symptoms in 14 of the 27 cases, improvement of pre-existent paresis in 7 cases, but deterioration of motor function in 6. Motor deterioration could be attributed to postoperative swelling phenomena in 5 of the 6 cases and was regressive within 72 hours under antiedemic therapy with 8 mg of dexamethasone orally administered 6 times a day. Only one case involved a longer-lasting high-grade brachial paresis (1/5). The follow-up CT revealed the cause to be local bleeding into the tumor cavity with a moderately space-occupying effect but direct involvement of the primary motor cortex. Conservative therapy led to gradual regression within 6 weeks.

#### Group C

Twenty cases (8.6%) showed significant potential changes with prolongation of latencies > 15% and reduction of amplitudes > 80%. These, in contrast to those in Group B, were no longer reversible despite their immediate effect on the microsurgical procedure. A direct correlation with the potential changes was found for the following surgical maneuvers:

(a) traction or pressure by a brain spatula (2/20),

(b) electrocoagulation near the primary motor cortex or motor pathways (5/20),

(c) microdissection between the tumor border and motor cortex (10/20) and

(d) displacement/shifting of the electrode strip (3/20).

Only the three cases of surgery-related electrode strip dislocation (with no possibility of adequate repositioning) were associated with postoperatively unchanged neurological symptoms. The other 17 cases with intraoperative occurrence of irreversible potential changes also evidenced postoperative deterioration of motor function by at least two BMRC grades, which was unchanged at the 3- and 6-month follow-up. Postoperative CTs and MRIs ruled out brain edema, infarction or bleeding in these cases but documented tumor removal with direct affection of Brodman Area 4.

## Discussion

Monopolar anodal cortical stimulation (MCS) for the intraoperative application under general anesthesia was first described by Taniguchi et al. [17] in 1993. With this stimulation technique they were able to induce muscle action potentials in the trunk and extremities, so-called compound muscle action potentials (CMAPs) that can be qualitatively analyzed for intraoperative cortical mapping and patient monitoring [4-6,17,21]. For Taniguchi et al. muscle activity recording seemed suitable especially for intraoperative monitoring as it can be recorded without causing obvious movement of the patient (which might be especially meaningful during microneurosurgery), as well as its potential size (which allows recording without averaging) and its latencies (which might supply the surgeon with quantitative and qualitative information about the motor system's integrity) [17]. The physiological basis of such motor effects following a transient stimulus to the cerebral cortex is in detail described by Amassian et al. [22] in the animal model, showing that the response to a surface stimulus applied to Brodman area 4 is a direct (D-) wave conducted in fast axons followed by several indirect (I-) waves if recorded from the cortico-motoneural cord and a specific motor action potential if recorded from certain muscle groups [22]. With an anodal stimulus applied to the cortex, current is assumed to enter at the apical dendrites, leading to depolarization at the proximal Ranvier internodes of the corticospinal tract axons [22]. Unfortunately, little is still known concerning the effect of the total charge and the total charge density of a number of pulses in train on the cortex excitability. One major concern is, that far field depolarization and current spread are more likely to occur with this technique. Therefore, MCS monitoring differs from the bipolar stimulation technique in that action must be taking immediately when potential changes are observed, assuming that they occur before motor function is damaged irreversibly, whereas repetitive bipolar mapping gives a more spatial information, e.g. on the anatomical localization of the motor pathways, allowing the surgeon to define margins which have to be preserved around the motor sites.

The success rate of MCS mapping was 97% in the 58 cases presented by Cedzich et al. in 1996 [20]. In the present study, CMAPs could be recorded after high-frequency anodal MCS in 91% of the 255 cases. This confirms the applicability of the method, which appears to have limitations only in children under the age of 2 (attributed to the still incomplete myelinization of the pyramidal tract) and in patients with pre-existent high-grade paresis (BMRC grade 2/5 or worse), whereas the presence and duration of a pre-existing preoperative paresis BMRC grade 3/5 or better has no significant influence on repetitive MCS as a monitoring procedure [23].

A frequency of 400 Hz combined with a train of 5 impulses and an impulse duration of 0.3 ms was most often applied successfully in our study. This preferred setting is comparable to that reported by Taniguchi et al. [17] and Cedzich et al. [19,20]. The mean stimulation intensity of 16.4 ± 6.7 mA required to trigger a CMAP with this combination of stimulation parameters was clearly below the upper safety threshold postulated by Agnew and McCreery [24]. In some cases the stimulation intensity could even be reduced as needed by increasing the frequency from 400 to 500 Hz or the train count from 5 to 7. Increasing the stimulation frequency or the train probably leads to a greater accumulation of EPSPs and thus ultimately to depolarization of motoneurons at a lower stimulation intensity [25,26]. Pulse duration does not seem to be an important factor in MCS. Pulses lasting 200–300 μs were sufficient in most of the cases. The use of longer pulses may unnecessarily increase the cortical load.

Muscle action potentials could be recorded most frequently from the upper extremity (thenar muscles and forearm flexors). The reason for this seems to be the larger representation field of the hand and forearm in the primary motor cortex [27]. Depending on the exact location of the target lesion, additional muscles, such as the orbicularis oris muscle of the face, may be included in the recording scheme. However, in the author's experience, recordings from the limbs picked up basically all motor impairment that could be found on postoperative examination. Since surgery-related displacement of the stimulation electrode can occur, however, it proved advantageous to apply a fixed installation pattern for the recording electrodes, which in each case involved an additional pair of subdermal needle electrodes over the quadriceps femoris and gastrocnemius muscles.

The typical CMAP is a polyphasic potential ranging between 10 μV and 10 mV of amplitude, occurring 15–25 ms (arm) or 25–35 ms (leg) post stimulus with a duration of 10–15 ms. The latency depends on the recording site and varies greatly between individuals. All 3 parameters (latency, potential width and amplitude) showed wide intra- and interindividual variation. Cedzich et al. reported a comparably high range for both cortex [20] and brain stem stimulation [19]. This may be due to the sometimes inaccurate placement of the stimulation electrode over the motor cortex. It remains to be clarified whether the electrical stimulation can lead to excitation of inhibitory as well as excitatory fibers, which would explain the intermittent occurrence of latency changes. The previously described excitation mechanism of monopolar cortex stimulation accounts for this, because here the electrical stimulus leads to depolarization of the pyramidal cell axons, which triggers an EPSP at the synapse of the first neuron. From that point on, stimulus conduction is independent of the intensity of the stimulus applied.

Another reason for the high variation could lie in the aesthetic procedure. Though a standardized aesthetic protocol was used in the present study, Angel [28], Calancie [29] and Sloan [30] have shown that the latencies can be influenced by individual reactions to the aesthetic applied or its blood concentration.

Apart from the interindividual differences in MCS mapping, individual potential fluctuations were also observed during MCS monitoring. Nonquantifiable concomitant stimulation of inhibitory components may be assumed as a possible explanation for the slightly fluctuating measurements („oscillation" around the basal value t_0_), especially for the latencies. In the course of repetitive measurements, the electrode may also be shifted mechanically or have its contact to the brain surface changed by rinsing fluid, blood or air, which can cause further fluctuations and potential changes without any pathological background.

Of the 3 parameters observed, the amplitudes showed the greatest variability. Spontaneous amplitude fluctuations of up to 50% were observed. This was attributed to the same mechanism already described for the latencies.

The evaluation of the individual potential widths disclosed both wide variations of up to 30% range around the t_0 _value but also considerable inconsistency. This is due to the recording of both monophasic and polyphasic response potentials that are independent of the intensity of direct cortex stimulation. Thus the authors do not consider the potential width to be a suitable intraoperative course parameter.

Correlation between potential changes and postoperative clinical symptoms: Twelve cases showed postoperative motor deficits despite uneventful intraoperative measurements. In 10 of the 12 cases, however, they were restricted to the first 72 hours after surgery. The follow-up CT showed postoperative brain edema in these cases. The positive effect achieved by intensified antiedematous therapy confirmed that these cases did not involve intraoperatively measurable substance damage. If the potentials thus remained unchanged until the end of the operation, it was possible to make a prognostic statement shortly after surgery. A sudden and complete signal loss within two successive measurements limits the informational value – a technical problem (e.g., electrode dislocation) must be excluded – and thus necessitates systematic error detection in the setup.

Permanent motor deficits (clinically unchanged on 3- and 6-month follow up) occurred in 19/232 cases (8.2%) of this study. Two cases (2/232, 0.9%) with uneventful MCS were caused by territorial infarction, in the other 17 cases (17/232, 7.3%) abnormal MCS was to be noticed during the phase of lesion resection. MCS was irreversible in all of these cases. Post-operative control CTs demonstrated total tumor resection within the anatomical and electrophysiologically confirmed precentral gyrus in 15 of the 17 cases. In comparison, Neuloh and Schramm [31] report about 9% new permanent deficits in a group of 140 central and insular space-occupying lesions, operated on under direct monopolar electrocortical stimulation, if only the monitored muscle groups and limbs are considered. This perfectly demonstrates the ethical dilemma between preserving function and the goal of total tumor resection, as this might correlate with a better survival rate.

However, damage to neural structures during brain tumor surgery can only be prevented if appropriate measures are taken while functional changes are reversible. That is why several authors [32-34] started using subcortical stimulation in addition to cortical mapping and monitoring. A number of high-quality publications give prove of the reliability of this intraoperative neurophysiological tool, although its limited specificity, its lack of quantifiable results and continuous monitorability seem to be a drawback of that method in the hand of the inexperienced user [31]. Keles et al [32], using bipolar cortical and subcortical stimulation in a group of 294 cases, calculated the risk of permanent motor deficits to be 7.6% if both stimulation sites demonstrated that the lesion was located within or adjacent to motor tracts. Noteworthy, the risk of permanent deficit decreased significantly in their study (down to 2.3%) when subcortical pathways could not be identified but cortical stimulation confirmed a functionally intact status – demonstrating, that eloquent cortex sites were close but not in direct contact with the lesion (>2–3 mm distance [31]). In a recently published paper by Eisner et al. [33] the authors report a 10% morbidity (1/10) if the lesion was found within the primary motor cortex and close to the pyramidal fiber tract. Post-operative CT- and MRI-scan verified radical tumor resection in all of their cases.

In conclusion, surgical morbidity for lesions immediately within the precentral gyrus or with direct contact to subcortical motor pathways seems to be dependent on more than the location and the intraoperative monitoring technique used alone. Other factors such as tumor histology (metastases vs. gliomas), surrounding edema or aggressiveness of tumor resection play an important role in the outcome as well. A detailed multivariate meta-analysis should give more information on this important topic. Furthermore, studies combining MCS with subcortical stimulation techniques (such as already performed with bipolar stimulation techniques [32-34]) should investigate the potential of subcortical mapping and/or monitoring in reducing the rate of permanent morbidity for operations in these high-risk eloquent motor areas.

## Conclusion

MCS must be considered a stimulation technique that enables reliable qualitative analysis of the recorded potentials, which may thus be regarded as directly predictive. However, there is no statistical prove that MCS can be used to quantify or validate the grade of paresis.

Having performed a detailed analysis of the 232/255 monitoring cases, the authors are of the opinion that a latency prolongation of > 15% and/or an amplitude reduction of > 80% should be established as significant potential changes requiring action.

Nevertheless, like other intraoperative neurophysiological examination techniques, MCS has technical, anatomical and neurophysiological limitations. A variety of surgical and non-surgical influences can be reason for false positive as well as false negative measurements.

## Abbreviations

BMRC – British medical research council

CMAP – Compound muscle action potential

CT – Computed tomography

EPSP – Excitatory postsynaptic potential

MCS – Monopolar cortex stimulation

MEP – Motor evoked potential

MRI – Magnetic resonance imaging

TIVA – Total intravenous anesthesia

TOF – Train of five

## Competing interests

The author(s) declare that they have no competing interests.

## Authors' contributions

OS – has made major contributions to conception and study design. He has been involved in collecting, analyzing and interpreting the data.

SS – has made substantial contributions to conception and study design and has been involved in revising it critically.

MB – has revised the manuscript critically for important intellectual content.

TK – has been involved in collecting and interpreting the data. He has revised the manuscript critically for important intellectual content and has given final approval of the manuscript to be published.
